# Neuroprotective Effect and Antioxidant Potency of Fermented Cultured Wild Ginseng Root Extracts of *Panax ginseng* C.A. Meyer in Mice

**DOI:** 10.3390/molecules26103001

**Published:** 2021-05-18

**Authors:** Chul-Joong Kim, Hyeon-Yeol Ryu, Somin Lee, Han-Joo Lee, Yoon-Soek Chun, Jong-Kyu Kim, Chang-Yeon Yu, Bimal Kumar Ghimire, Jae-Geun Lee

**Affiliations:** 1 Research Institute of Biotechnology, HwajinBioCosmetics CO., LTD, Chuncheon 24232, Korea; kjjup2@naver.com; 2 Korea Conformity Laboratories, Yeonsu, Incheon 21999, Korea; rhyckato98@kcl.re.kr (H.-Y.R.); somin14@kcl.re.kr (S.L.); 3 Aribio H&B CO.LTD, Yongin 16914, Korea; hjlee@aribiohnb.com (H.-J.L.); yschun@aribiohnb.com (Y.-S.C.); jkkim@aribiohnb.com (J.-K.K.); 4 Department of Bio-Resource Sciences, Kangwon National University, Chuncheon 21341, Korea; cyyu@kanwon.ac.kr; 5 Department of Crop Science, College of Sanghuh Life Science, Konkuk University, Gwangjin, Seoul 05029, Korea; bimal_g12@yahoo.com

**Keywords:** cultured wild ginseng roots, *Pediococcus pentosaceus*, ginsenosides, acetylcholinesterase, memory deficit, antioxidant

## Abstract

Wild ginseng has better pharmacological effects than cultivated ginseng. However, its industrialization is limited by the inability to grow wild ginseng on a large scale. Herein, we demonstrate how to optimize ginseng production through cultivation, and how to enhance the concentrations of specific ginsenosides through fermentation. In the study, we also evaluated the ability of fermented cultured wild ginseng root extract (HLJG0701-β) to inhibit acetylcholinesterase (AChE), as well as its neuroprotective effects and antioxidant activity. In *in* *vitro* tests, HLJG0701-β inhibited AChE activity and exerted neuroprotective and antioxidant effects (showing increased catalyst activity but decreased reactive oxygen species concentration). In *in* *vivo* tests, after HLJG0701-β was orally administered at doses of 0, 125, 250, and 500 mg/kg in an animal model of memory impairment, behavioral evaluation (Morris water maze test and Y-maze task test) was performed. The levels of AChE, acetylcholine (ACh), blood catalase (CAT), and malondialdehyde (MDA) in brain tissues were measured. The results showed that HLJG0701-β produced the best results at a dose of 250 mg/kg or more. The neuroprotective mechanism of HLJG0701-β was determined to involve the inhibition of AChE activity and a decrease in oxidative stress. In summary, both *in* *vitro* and *in* *vivo* tests confirmed that HJG0701-β administration can lead to memory improvement.

## 1. Introduction

Dementia is a symptom of neurodegenerative diseases including Alzheimer’s disease (AD) [1]. Typical symptoms of AD include memory loss, depression, lapses in judgment, confusion, and cognitive loss [2,3]. Oxidative stress, metal ion homeostasis, mitochondrial dysfunction, neuroinflammation, apoptosis, endoplasmic reticulum dysfunction, and cell cycle imbalance have been implicated in the development of AD [2,4]. There is mounting evidence that damage to neuronal synapses, loss of neuronal function, decreased neuronal metabolic function, and damage to neurotransmitter systems can be caused by excessive deposition of amyloid-β_25–35_ (Aβ_25-35_) protein, a representative neuropathological cause of AD, in brain cells [2,5,6]. Aβ_25-35_ accumulates in cells due to the effects of reactive oxygen species (ROS), such as hydrogen peroxide (H_2_O_2_) generated *in vivo* through metabolic processes, superoxide anions, hydroxyl radicals, etc., which can cause DNA damage, protein oxidation, and lipid peroxidation, resulting in cell dysfunction, the collapse of cell membrane fluidity, and apoptosis, leading to AD [7].

The degradation of ACh, a neurotransmitter, by AChE can lead to declines in cognitive function and memory as AChE plays an important role in learning and memory [8]. Efforts are underway to activate the cholinergic system in the brain using a cholinergic agonist or AChE inhibitor that can inhibit ACh hydrolysis, with the aim of alleviating the deficiency in cholinergic neurons by inhibiting AChE activity [9]. Donepezil, rivastigmine, galantamine, and memantine are typical therapeutic drugs used in AD clinical trials. All these therapeutic drugs, except memantine, an *N-*methyl-d-aspartate (NMDA) receptor antagonist, are cholinergic inhibitors that act in the brain [10]. Studies have shown that cholinergic action is a key factor in AD [11,12]. However, the AChE inhibitors that are used to improve the cognitive function of patients with AD are associated with numerous side effects, including nausea, diarrhea, anorexia, and abdominal pain [11]. Accordingly, researchers are attempting to develop AChE inhibitors derived from natural products that can replace the existing AChE inhibitors [13].

*Panax ginseng* C.A. Meyer, which belongs to the family Araliaceae, is naturally distributed in Korea, China, Japan, Europe, and North America [14,15,16]. The roots of these plants are traditionally used as herbal medicines for the treatment of human diseases [17], while certain plant extracts are used to improve immune system and liver function [18,19]. The root extracts of this plant have also been shown to possess anti-stress, anti-diabetic, anti-inflammatory, antioxidative, anti-aging, anti-cancer, and immunomodulatory activity, along with neuroprotective, anti-fatigue, cardio-protective, and hepato-protective physiological and pharmacological effects [20,21,22,23]. Previous studies have reported the presence of numerous phytochemicals in ginseng roots, including ginsenosides, flavonoids, monoterpenes, triterpenes, fatty acids, phenylpropanoids, alkanes, alkynes, sterols, kairomones, and polysaccharides [24,25]. Among these bioactive compounds, ginsenosides are the most important due to their wide range of health benefits, which include cancer prevention and immunomodulatory activity [26]. Previous studies have shown that the consumption of ginseng rich in ginsenoside contributes significantly to preventing health problems, including a reduction in cerebral ischemic injury [27], improved levels of anti-apoptosis mediators in the rat brain [28], and improved the Aβ-induced mitochondrial pathology [29].

The conventional methods of growing ginseng in the cultivation field are time-consuming and labor-intensive, which translates into increased costs [30]. The use of bioreactors for the cultured wild ginseng roots is beneficial and economical for the production of valuable bioactive compounds [31,32]. The technology for producing ginsenosides using the cultured wild ginseng roots, after introducing wild ginseng hair cells, offers the advantages of genetic safety and high productivity compared to using wild ginseng in its natural habitat. Such technology is already being used in the development of functional foods and physiologically active products [33,34]. A number of previous studies have shown that the high-molecule ginsenosides in ginseng (molecular weight of 947.15–1109.29) cannot be absorbed in the human intestine [35]. To decompose the sugars bound to high-molecule ginsenosides, physical and chemical processes such as acid treatment, heat treatment (steaming), and fermentation are used to promote conversion into small-molecule ginsenosides [36]. Decomposed small-molecule ginsenosides can be effectively absorbed by the body and are pharmacologically active [37]. Rg3, Rk1, and Rg5 are three typical small-molecule ginsenosides [38]. Rg3 can reportedly lower blood pressure and exert anti-cancer effects [39,40]. Rg3, Rk1, and Rg5 can prevent dementia [41,42,43] and osteoporosis [44]. Furthermore, Compound K has anti-inflammatory [45] and hepato-protective effects [46]. Tissue culture technology, one of the technologies of plant production, enables the differentiation of cells into tissues or organs by the totipotency of plants, therefore it is at the center of researches into plant systems capable of producing specific metabolic products. Through cells and callus suspension culture, it has the advantages of genetic and biochemical stability, hormone autotrophy, and multienzyme biosynthetic capacity, etc., as a biocatalyst [47,48]. The synthesis of metabolites and biotransformation by an “elicitor” can bring about changes in subjects for research in substances originating from plants, which are realistically unavailable. Unprecedented plant molecules belonging to a completely new group, available for commercial utilization including therapeutic purposes, were discovered in the process of biotransformation, by which the use of hazardous chemical substances can be avoided, and accordingly, it is spotlighted as an initial step in the field of green chemistry [49].

The objective of the present study is to optimize ginseng production through *in vitro* culturing and to enhance the contents of specific ginsenosides in fermented cultured wild ginseng roots using HPLC analysis. We also evaluated the ability of a fermented cultured wild ginseng roots extract, HLJG0701-β, to inhibit AChE activity and assessed its neuroprotective and antioxidant effects using a scopolamine and ovariectomized (OVX) + d-galactose-induced animal model of aging and memory impairment.

## 2. Results

### 2.1. Ginsenoside Content

**Figure 1:**
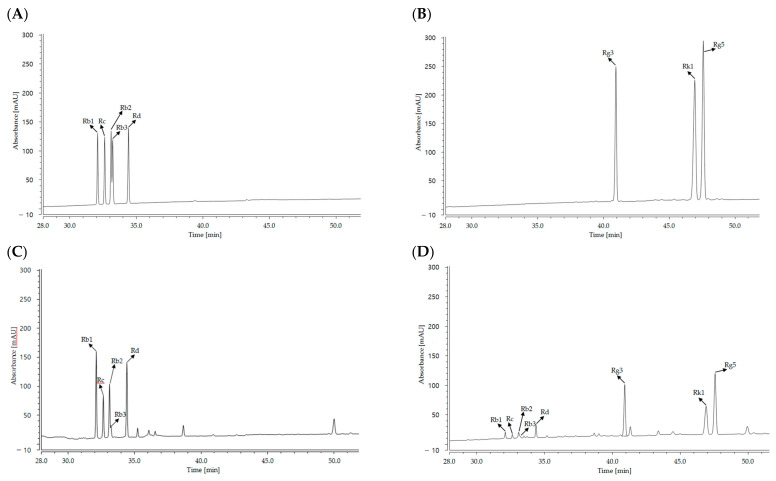
Representative HPLC chromatograms of ginsenosides detected incultured wild ginseng root and HLJG0701-β. (**A**) Ginsenosides Rb1, Rc, Rb2, Rb3, and Rd reference standards; (**B**) ginsenosides Rg3, Rk1, and Rg5 reference standards; (**C**) cultured wild ginseng root; (**D**) HLJG0701-β.

Quantitative analysis was performed on eight types of ginsenosides, divided into high-molecule and small-molecule ginsenosides. The total concentration of ginsenosides in cultured wild ginseng root treated with jasmonate was 188.06 ± 4.98 mg/g. Five high-molecule ginsenosides (Rb1, Rc, Rb2, Rb3 and Rd) were detected, but no small-molecule ginsenosides (Rg3, Rk1, and Rg5) were detected. The total concentration of ginsenosides in fermented HLJG0701-β was 117.96 ± 3.38 mg/g. The levels of high-molecule ginsenosides (Rb1, Rc, Rb2, Rb3, and Rd) were decreased in this specimen compared to the unfermented specimen, while the levels of small-molecule ginsenosides were detectable (Rg3, 44.26 ± 1.02 mg/g; Rk1, 15.93 ± 0.32 mg/g; Rg5, 23.10 ± 0.59 mg/g) in the fermented sample (Table 1, Figure 1). Overall, the high-molecule ginsenoside concentrations were altered by fermentation, such that the contents of Rg1 (51.53 ± 1.34 mg/g→9.26 ± 0.28 mg/g), Rc (38.16 ± 1.10 mg/g→4.93 ± 0.41 mg/g), Rb2 (34.36 ± 1.26 mg/g→6.36 ± 0.40 mg/g), Rb3 (8.10 ± 0.52 mg/g→2.83 ± 0.35 mg/g), and Rd (55.90 ± 0.85 mg/g→11.26 ± 0.56 mg/g) tended to decrease. In addition, the small-molecule ginsenosides (Rg3, Rk1, and Rg5), which were not present in the cultured wild ginseng root treated with methyl jasmonate, accounted for 83.29 mg/g of all ginsenosides after fermentation. Table 1 and Figure 1 illustrate the changes in ginsenoside content that occurred during fermentation. These increases in the concentrations of ginsenosides Rg3, Rk1, and Rg5 are thought to be due to the fermentation-induced conversion of high-molecule ginsenosides to small-molecule ginsenosides.

### 2.2. In Vitro Experiment

#### 2.2.1. Neuroprotective Effect

##### AChE Activity

**Figure 2:**
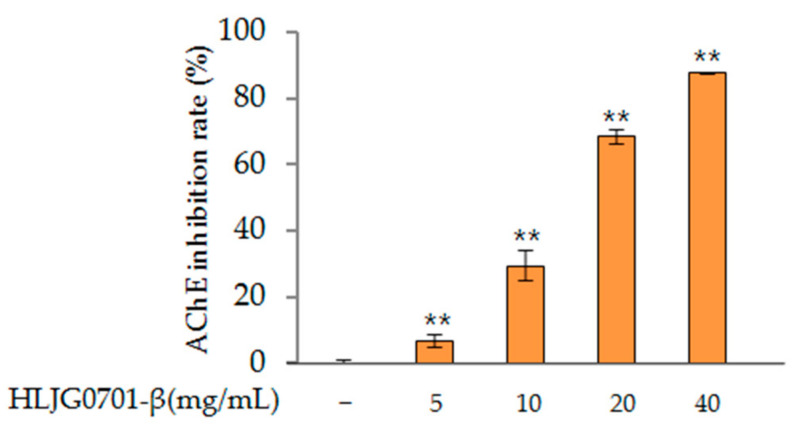
AChE inhibition rates of HLJG0701-β. Data are presented as mean ± standard deviation. Error bar: standard deviation; **, significant difference compared with control by one-way ANOVA, *p* < 0.01.

To determine the potential utility of HLJG0701-β, its inhibitory potency was evaluated according to a modified Ellman procedure. Initially, all obtained HLJG0701-β specimens were tested at concentrations of 5 mg/mL and 40 mg/mL. HLJG0701-β showed more than 60% activity at concentrations of 20 mg/mL (68.56 ± 2.12%) and 40 mg/mL (87.65 ± 0.16%) (*p* < 0.01). Since HLJG0701-β exhibited AChE inhibitory effects, it may be able to enhance memory and prevent dementia, suggesting the possibility of its application as a therapeutic AChE inhibitor (Figure 2).

##### Nerve Cell Damage-Induced Protective Effect

**Figure 3:**
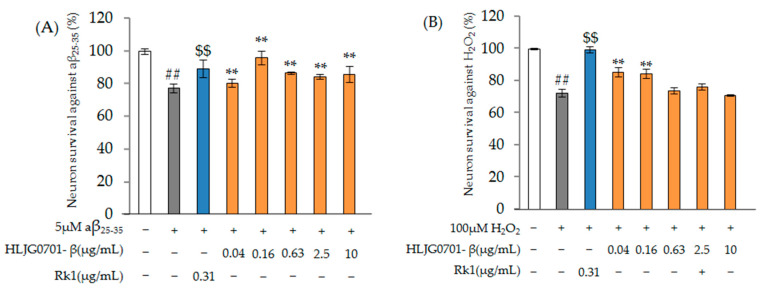
Neuroprotective effects against (**A**) amyloid-β_25-35_ and (**B**) H_2_O_2_ induced damage. Data are presented as mean ± standard deviation. Error bar: standard deviation; ##, significant difference compared with control by Student’s *t*-test, *p* < 0.01; $$, significant difference compared with negative control by Student’s *t*-test, *p* < 0.01; **, significant difference compared with negative control by one-way ANOVA, *p* < 0.01.

Aβ_25-35_ and H_2_O_2_ were separately added to SH-SY5Y cells (5 × 10^4^cells/96-well), which were then incubated at 37 °C for 24 h, to investigate the potential neuroprotective effects of the ginsenosides. In the Aβ_25-35_ test, the treatment of SH-SY5Y cells with 5 µM Aβ_25-35_ alone led to a significant decrease in cell viability, to 77.31 ± 2.74%, versus treatment with 0.31 µg/mL ginsenoside Rk1 positive control (89.29 ± 5.62%) (*p* < 0.01). The treatment group showed more than 80% activity at concentrations of 0.04 µg/mL (82.48 ± 0.51%), 0.16 µg/mL (80.52 ± 2.45%), 0.63 µg/mL (96.01 ± 4.04%), 2.5 µg/mL (84.23 ± 1.51%), and 10 µg/mL (86.05 ± 4.96%) (*p* < 0.01) (Figure 3A).

In the H_2_O_2_ test, the treatment of SH-SY5Y cells with 100 µM H_2_O_2_ alone led to a significant decrease in cell viability, to 72.61 ± 2.38%, versus treatment with 0.31 µg/mL ginsenoside Rk1 positive control (99.58 ± 3.63%) (*p* < 0.01). In the test in which oxidative stress was induced by treatment with H_2_O_2_, when a low concentration of HLJG0701-β was treated, it was confirmed that SH-SY5Y cells were statistically significantly protected by the test substance when compared to the control group (Figure 3B). Even at low concentrations, HLJG0701-β showed significant protective effects against the nerve cell damage caused by toxic Aβ_25-35_ and H_2_O_2_. Therefore, HLJG0701-β has a protective effect against neuronal damage.

#### 2.2.2. Antioxidant Effect

##### ROS and CAT Activity

HLJG0701-β was then pretreated, followed by incubation at 37 °C for 24 h. ROS production was induced in SH-SY5Y cells (2 × 10^6^ cells/6-well) by treatment with H_2_O_2_ for 4 h. After staining with DCFDA, fluorescence expression was measured to compare ROS activity between samples.

**Figure 4:**
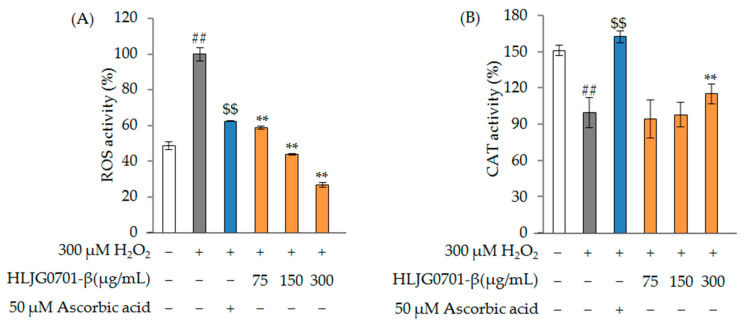
Effect of HLJG0701-β on antioxidant (**A**) ROS (**B**) CAT activity. Data are presented as mean ± standard deviation. Error bar: standard deviation; ##, significant difference compared with control by Student’s *t*-test, *p* < 0.01; $$, significant difference compared with negative control by Student’s *t*-test, *p* < 0.01; **, significant difference compared with negative control by one-way ANOVA, *p* < 0.01.

As expected, H_2_O_2_ induced oxidative stress, as ROS activity was significantly increased following treatment with 300 µM H_2_O_2_ compared to the control (*p* < 0.01). The test group treated with positive control (300 µM H_2_O_2_ + 50 µM ascorbic acid) showed a ROS activity level of 62.51 ± 0.31% versus the H_2_O_2_ treatment group (100%). All treatment groups showed a reduction in H_2_O_2_-induced ROS activity (300 µM H_2_O_2_ + 75, 150, 300 µg/mL HLJG0701-β) = (58.85 ± 0.85%, 43.77 ± 0.52%, 26.9 ± 1.37%) (Figure 4A) (*p <* 0.01).

CAT activity was significantly decreased in the 300 µM H_2_O_2_ treatment group compared to the control (*p* < 0.01). The test group treated with positive control (300 µM H_2_O_2_ + 50 µM ascorbic acid) showed 163.04 ± 4.82% of CAT activity versus the H_2_O_2_treatment group (*p <* 0.01). All treatment groups showed changes in H_2_O_2_-induced CAT activity (300 µM H_2_O_2_ + 75, 150, 300 µg/mL HLJG0701-β) = (94.9 ± 16.03, 98.43 ± 9.92, 115.69 ± 8.16%) (Figure 4B) (*p* < 0.01).

### 2.3. In Vivo Experiment

#### 2.3.1. Scopolamine-Induced Animal Model

##### Morris Water Maze Task

The underwater maze test is widely used to measure hippocampal-dependent spatial perception and learning ability. When memory and learning ability are preserved, the time to reach the stable platform (escape latency) is reduced.

**Figure 5:**
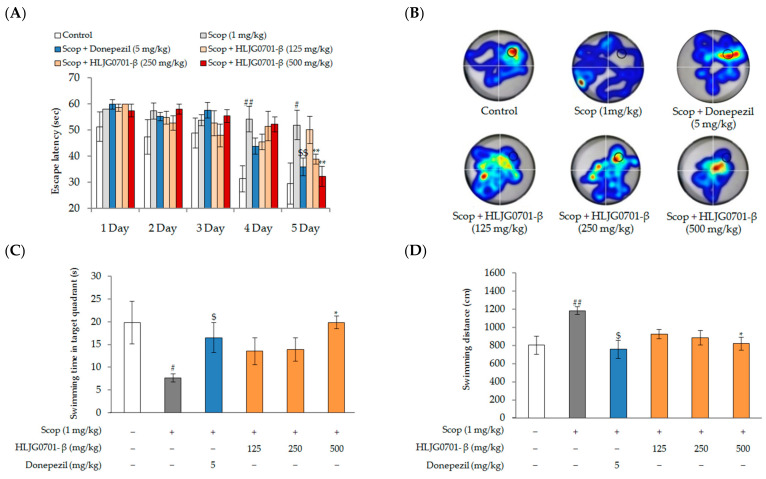
The Morris Water Maze task in scopolamine-induced male mice. (**A**) escape latency; (**B**) heatmap images; (**C**) swimming time in target quadrant; (**D**) swimming distance. Data are presented as mean ± standard deviation. Error bar: standard deviation. ##, significant difference compared with control by Student’s *t*-test, *p <* 0.01; #,significant difference compared with control by Student’s *t*-test, *p <* 0.05; $$,significant difference compared with scopolamine group by Student’s *t*-test, *p* < 0.01; $, significant difference compared with scopolamine group by Student’s *t*-test, *p* < 0.01; **,significant difference compared with scopolamine group by one-way ANOVA, *p <* 0.01; *,significant difference compared with scopolamine group by one-way ANOVA, *p <* 0.05.

The performance of all groups in the MWM training task is shown in Figure 5. The behavior of mice in all groups improved during successive training days, as evidenced by shortened escape latencies. The scopolamine group took longer to find the platform than the control group, from the second day to the fifth day of the training period, showing that scopolamine treatment resulted in significant cognitive dysfunction in mice (*p* < 0.01, *p* < 0.05). Meanwhile, 250 and 500 mg/kg HLJG0701-β and 5mg/kg donepezil treatment significantly increased the escape latency on the fourth (51.6 ± 5.7 s, 52.4 ± 2.9 s, 44.0 ± 4.9 s, respectively) and fifth (39.0 ± 1.9 s, 32.4 ± 3.8 s, 36.1±5.6s, respectively) (*p* < 0.01) days of the training period, compared to the scopolamine group (54.3 ± 3.1 s, 52.1 ± 3.4 s, respectively) (*p* < 0.01, *p* < 0.05). The swimming time in the target quadrant of the scopolamine group (7.7±0.9 s) was significantly less than that of the control group (19.8 ± 4.7 s) (*p* < 0.05). On the other hand, the swimming time in the target quadrant was extended in the positive control treatment groups (16.5 ± 3.3 s, 13.6 ± 3.0 s, 13.9 ± 2.6 s, 19.9 ± 1.4 s, respectively). Although the differences were not significant, an increased swimming time was observed in mice exposed to 125 and 250 mg/kg HLJG0701-β (Figure 5B,C). As shown in Figure 5D, the total swimming distance of each group decreased. The distance traveled by mice in the positive control group decreased throughout the training period (758.1 ± 98.0 cm) (*p* < 0.05). In addition, the swimming distance of the treatment groups (925.1 ± 52.9 cm, 886.6± 81.9 cm, 821.5 ± 72.6 cm, respectively) was clearly less than that of the scopolamine group (1184.6 ± 42.0 cm).

##### Y-Maze Task

**Figure 6:**
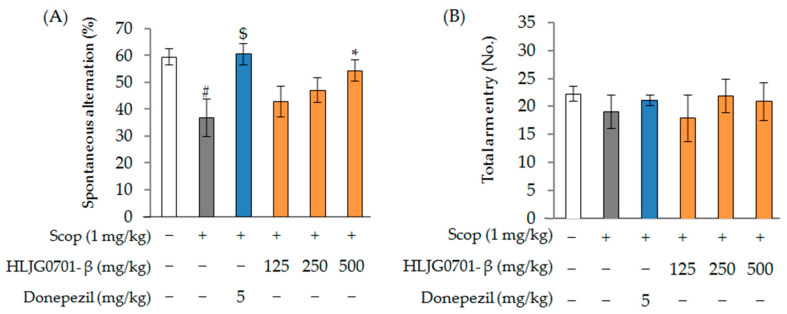
The Y-maze task in scopolamine-induced male mice. (**A**) Spontaneous alternation; (**B**) Total arm entry. Data are presented as mean ± standard deviation, Error bar: standard deviation. #, significant difference compared with control by Student’s *t*-test, *p <* 0.05; $, significant difference compared with scopolamine group by Student’s *t*-test, *p* < 0.01; *, significant difference compared with scopolamine group by one-way ANOVA, *p <* 0.05.

Scopolamine was used to produce AD-like memory deficits in mice, and donepezil (5 mg/kg, positive control), an approved AChE inhibitor, was employed as a positive reference drug. Figure 6A shows the effects of scopolamine and HLJG0701-β (125, 250, 500 mg/kg) treatment on spontaneous alternation behavior and locomotor activity (total arm entry) in the Y-maze test. Scopolamine injection significantly decreased short-term memory performance, as evidenced by the decrease in spontaneous alternation percentage compared with the control group (*p* < 0.05). Spontaneous alternation significantly decreased to 37.1 ± 7.1% in the scopolamine group, from 59.8 ± 3.1% in the control group. However, the scopolamine-induced decrease in the spontaneous alternation rate was significantly increased in the positive control group (60.8 ± 3.9%) (*p* < 0.05), 125 and 250 mg/kg HLJG0701-β treatment groups (43.1 ± 5.6%, 47.4 ± 4.5%, respectively), and 500 mg/kg HLJG0701-β treatment group (54.7 ± 4.1%) (*p* < 0.05).

The total number of entrances and exits for each arm of the maze was measured for all test animals; no statistically significant difference was observed among all test groups (Figure 6B).

#### 2.3.2. Ovariectomized (OVX) + d-Galactose-Induced Animal Model

##### Morris Water Maze Task

**Figure 7:**
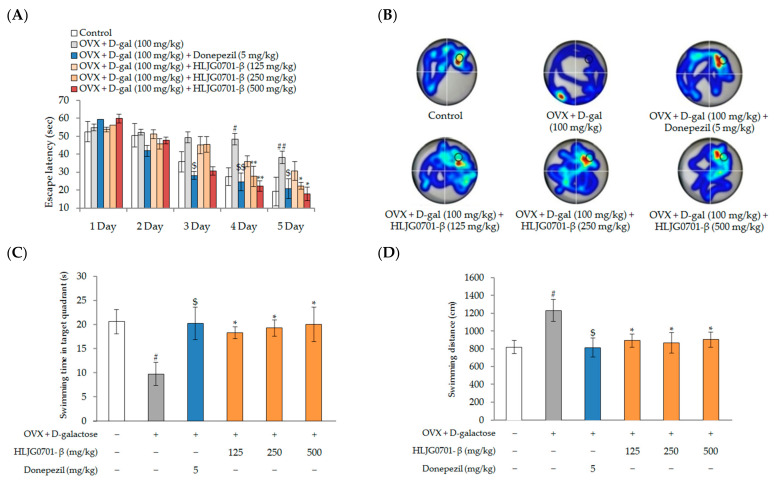
The Morris Water Maze task in OVX + d-galactose-induced female mice. (**A**) Escape latency; (**B**) heatmap images; (**C**) swimming time in target quadrant; (**D**) swimming distance. Data are presented as mean ± standard deviation. Error bar: standard deviation. ##, significant difference compared with control by Student’s *t*-test, *p <* 0.01; #, significant difference compared with control by Student’s *t*-test, *p <* 0.05; $$, significant difference compared with OVX + d-galactose group by Student’s *t*-test, *p* < 0.01; $, significant difference compared with OVX + d-galactose group by Student’s *t*-test, *p* < 0.05; **, significant difference compared with OVX + d-galactose group by one-way ANOVA, *p <* 0.01; *, significant difference compared with OVX + d-galactose group by one-way ANOVA, *p <* 0.05.

The performance of all groups in the MWM training task is shown in Figure 7. The behavior of mice in all groups improved during successive training days, as evidenced by shortened escape latency. The ovariectomized (OVX)+ d-galactose group took more time to find the platform than did the control group from the fourth to the fifth day of the training period, showing that OVX + d-galactose-induced changes could cause significant cognitive dysfunction in mice (*p* < 0.01, *p* < 0.05). Meanwhile, the positive control treatment groups exhibited significant amelioration of the OVX + d-galactose treatment increase in escape latency on the third, fourth (36.1 ± 5.5 s, 27.8 ± 5.3 s, 22.3 ± 3.1 s, 24.6 ± 4.8 s, respectively) and fifth (30.8 ± 5.8 s, 22.4 ± 4.7 s, 18.0 ± 3.6 s, 20.9 ± 5.9 s, respectively) days of the training period compared to the OVX + d-galactose group (48.4 ± 4.5 s, 38.3 ± 3.3 s, respectively).

The swimming time in the target quadrant of the OVX + d-galactose group (9.8 ± 2.4 s) was significantly less than that of the control group (20.7 ± 2.5 s) (*p* < 0.05). On the other hand, the swimming time was extended in the positive control treatment groups (20.3 ± 3.4 s, 18.4 ± 1.2 s, 19.3 ± 1.7 s, 20.1 ± 3.6 s, respectively) (*p* < 0.05). Swimming time was significantly increased in mice exposed to the treatment group (Figure 7B,C).

As shown in Figure 7D, the total swimming distance of each group was decreased. The swimming distance in the positive control group decreased throughout the training period (819.4 ± 109.1cm) (*p* < 0.05). In addition, the swimming distance of the treatment groups (897.8 ± 75.0 cm, 872.6 ± 115.5 cm, 908.1 ± 86.5 cm, respectively) were clearly reduced in comparison with that of the OVX + d-galactose group (1234.9 ± 123.6 cm) (*p* < 0.05).

##### Y-Maze Task

**Figure 8:**
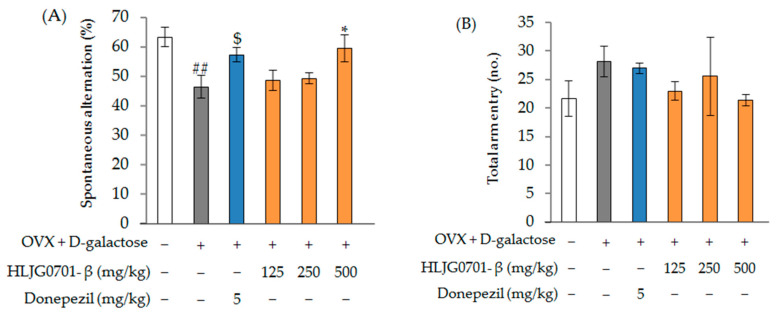
The Y-maze task in OVX + d-galactose-induced female mice. (**A**) Spontaneous alternation; (**B**) total arm entry. Data are presented as mean ± standard deviation. Error bar: standard deviation. ##, significant difference compared with control by Student’s *t*-test, *p* < 0.01; $, significant difference compared with OVX + d-galactose group by Student’s *t*-test, *p* < 0.05; *, significant difference compared with OVX + d-galactose group by one-way ANOVA, *p* < 0.05.

Figure 8A shows the effects of OVX + d-galactose and HLJG0701-β (125, 250, 500 mg/kg) treatment on spontaneous alternation behavior and locomotor activity (total arm entry) in the Y-maze test. OVX + d-galactose-induced mice showed a significant decrease in short-term memory performance, as evidenced by a decreased spontaneous alternation percentage compared with the control group (*p* < 0.01). Spontaneous alternation significantly decreased to 46.5 ± 3.9% in the OVX + d-galactose group, from 63.3 ± 3.3% in the control group. However, the OVX + d-galactose-induced decrease in the spontaneous alternation rate was significantly increased in the positive control group (57.4 ± 2.5%) (*p* < 0.05), treatment (HLJG0701-β 125, 250 mg/kg) groups (48.7 ± 3.5%, 49.3 ± 1.9%, respectively), and HLJG0701-β 500 mg/kg group (59.5 ± 4.6%) (*p* < 0.05). When the total number of entrances and exits for each arm was measured for each test animal, no statistically significant differences were observed among all test groups (Figure 8B).

##### Acetylcholinesterase (AChE) and Acetylcholine (ACh) Contents

**Figure 9:**
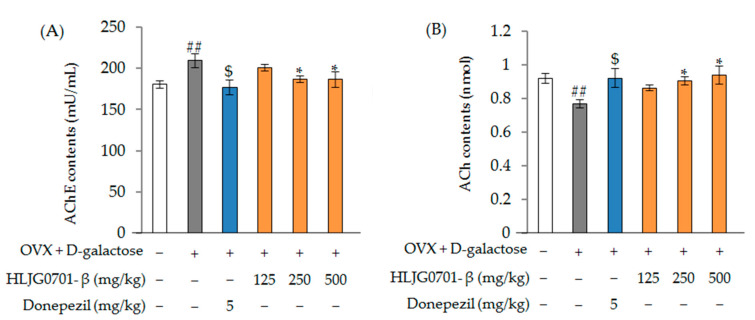
Effect of HLJG0701-β on the contents AChE (**A**) and ACh (**B**) in mice brain tissue. Data are presented as mean ± standard deviation. Error bar: standard deviation. ##, significant difference compared with control by Student’s *t*-test, *p <* 0.01; $, significant difference compared with OVX + d-galactose group by Student’s *t*-test, *p* < 0.05; *, significant difference compared with OVX + d-galactose group by one-way ANOVA, *p <* 0.05.

The effects of HLJG0701-β on brain AChE and ACh contents are shown in Figure 9A, B. AChE activity was significantly up-regulated in the OVX + d-galactose group (210.09 ± 8.06 mU/mL) (*p* < 0.01) compared to the control group (181.18 ± 4.73 mU/mL). When the OVX + d-galactose-induced mice were treated with HLJG0701-β (250, 500 mg/kg), a marked inhibition of AChE activity (187.44 ± 4.19 mU/mL, 187.11 ± 9.02 mU/mL) in the brain tissue was observed in comparison with the OVX + d-galactose group (*p* < 0.01). Furthermore, donepezil treatment (positive control group) significantly suppressed the increase in AChE activity (177.84 ± 9.20) (*p* < 0.05).

Meanwhile, ACh activity was found to be lower in the OVX + d-galactose group (0.769 ± 0.025 nmol) than in the control group (0.921 ± 0.028 nmol) (*p* < 0.01). In addition, the treatment (HLJG0701-β 125, 250 mg/kg) groups (0.906 ± 0.025, 0.942 ± 0.054 nmol) showed a clear reversal of the decrease in the OVX + d-galactose group (*p* < 0.05). These results indicate that administration of HLJG0701-β suppressed the increase in AChE activity and the reduction of ACh levels induced by OVX + d-galactose.

##### Malondialdehyde (MDA) and Catalase (CAT) Contents

**Figure 10:**
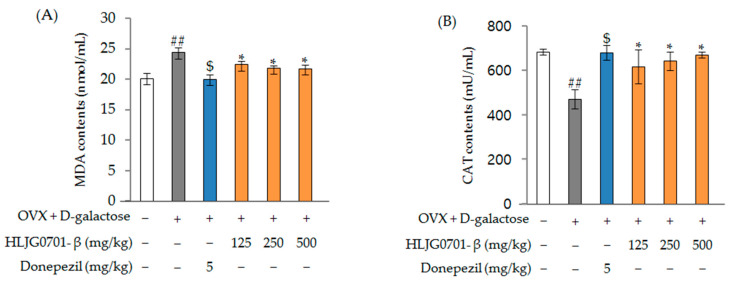
Effect of HLJG0701-β on the contents MDA (**A**) and CAT (**B**) in the blood of mice. Data are presented as mean ± standard deviation. Error bar: standard deviation. ##, significant difference compared with control by Student’s *t*-test, *p <* 0.01; $, significant difference compared with OVX + d-galactose group by Student’s *t*-test, *p* < 0.05; *, significant difference compared with OVX + d-galactose group by one-way ANOVA, *p <* 0.05.

Figure 10A shows the MDA concentration in blood. In the OVX + d-galactose group, OVX + d-galactose-induced changes significantly increased the MDA contents (24.40 ± 0.77 nmol/mL) compared to that in the positive control treatment (HLJG0701-β 125, 250, 500 mg/kg) groups (20.00 ± 0.80, 22.43 ± 0.59, 21.90 ± 0.37, 21.70 ± 0.66 nmol/mL, respectively) (*p* < 0.05). The changes in CAT activity in the blood tissues are shown in Figure 10B. CAT activity significantly decreased to 471.60 ± 42.87 mU/mL in the OVX + d-galactose group, from 684.00 ± 14.00 mU/mL in the control group (*p* < 0.01). However, the decrease in CAT contents induced by OVX + d-galactose was significantly ameliorated in the positive group (680.18 ± 35.66 mU/mL) and treatment (HLJG0701-β 125, 250, 500 mg/kg) groups (617.40 ± 75.87, 643.00 ± 40.66, 670.00 ± 14.28 mU/mL) (*p* < 0.05).

## 3. Discussion

The natural growth rate of wild ginseng is very low, making it difficult to commercialize. Luckily, cultured wild ginseng roots produced by *in vitro* culture are known to have a similar genetic DNA to wild ginseng, with high saponin contents [50,51]. In this study, we optimized an adventitious root culture of ginseng using a bioreactor [52]. In the present study, HPLC analysis of the fermented cultured wild ginseng roots extract of ginseng (HLJG0701-β) revealed the presence of increased concentrations of small molecular weight ginsenosides such as Rb1, Rc, Rb2, Rb3, and Rd, which may be attributable to the bacterial conversion of high molecular weight ginsenosides. Small-molecule ginsenosides are known to influence neurotransmission by a variety of mechanisms, including the regulation of synthase and signaling pathways of specific neurotransmitter systems as well as the release of neurotransmitters [53]. Among different ginsenosides, Rg3, Rk1, and Rg5 are known to improve Aβ_25-35_ levels, memory, and cognitive function [54].

Many previous studies have shown that ACh is required for learning and memory [55,56,57]. Impairments in memory, behavior, thinking, and judgment are closely related to diseases such as AD [58,59,60]. Clinically, AChE inhibitors such as donepezil are commonly used for the treatment of AD [60,61]. Several recent studies reported on the phytochemical-induced inhibition of AChE [62]. In the present study, *in vitro* AChE inhibitory activity was observed using increasing concentrations of HLJG0701-β. Donepezil, the positive control, inhibited AChE activity in a dose-dependent manner. The improvement in memory following treatment with HLJG0701-β was comparable to that produced by donepezil treatment. Interestingly, the AChE inhibition rate of HLJG0701-β at a concentration of 40 mg/mL was greater than that of donepezil, indicating that HLJG0701-β may enhance the production of ACh. Moreover, the ginseol K-g3, a constituent of ginseng roots, has been reported to reverse scopolamine-induced amnesia in treated mice [63]. A number of previous studies observed an increase in the uptake of choline in the cerebral nerve endings [64] and of enhanced acetylcholine (ACh) synthesis and uptake [65], which help to increase the memory and learning process. The ginsenosides such as Rg3, Rg5, Rg1, Rb1 and Rg1 are known for improving the level of choline acetyltransferase (ChAT) in mice brains [66,67] and decreased memory loss [67,68,69]. In other studies, Rg3 ginsenosides have shown neuroprotective activity against excitotoxicity [40,43,70]. It has been argued that ginseng extracts containing Rg3 enhanced long-term memory in mice through the cholinergic nervous system [71]. Moreover, it is possible that treatment of the mice with ginseng extracts may have reversed scopolamine-induced amnesia, due to actions on neurons in the basal region of the forebrain. Our results corroborate a previous report, which found a protective effect of wild ginseng extracts on scopolamine-induced amnesia [70,72]. In the present study, the higher concentration of Rg3 in HLJG0701-β than in wild ginseng may have been directly associated with the observed improvements in memory. Moreover, a number of previous studies suggested that the ginsenosides, including Rb1, Rg1, Rg5 and Rd, are associated with the reduction of cerebral Aβ in the mice brain and an increased memory and learning process [43,73,74,75,76,77,78]. The possible mechanism of attenuating Aβ aggregation in the brain takes place by enhancing the activity of α-secretase in the suppression of β-secretase [51,79]. Moreover, ginsenosides such as Rb1 and Rg1 are associated with protection against neurotoxic damage and the inhibition of α-synuclein aggregation in the brain [80,81,82,83,84]. Thus, HLJG0701-β containing ginsenoside compounds may be associated with improving the level of choline acetyltransferase in the brain and may protect against neuroprotective damage.

Neurodegenerative diseases such as AD and Parkinson’s Disease are caused by oxidative stress-induced cell damage [9,10,56]. Certain types of ROS, such as superoxide (O2^•−^) and hydroxyl (^•^OH), play a crucial role in inducing these disorders in the human body [56]. In the present study, the increased ROS levels were markedly reduced by HLJG0701-β at a concentration of 300 µg/mL, indicating that HLJG0701-β is a potent radical scavenger. OVX + d-galactose-induced changes significantly increased the MDA levels in the treated mouse brain. In the present study, HLJG0701-β significantly inhibited MDA levels at all concentrations, suggesting that HLJG0701-β has superior protective abilities, possibly because of its antioxidant properties.

Antioxidant enzymes such as CAT, GPx, and SOD, as well as non-enzymes such as glutathione and vitamins, are known to play important roles in intracellular ROS scavenging [85]. CAT is responsible for the decomposition of H_2_O_2_, which is essential for cellular signaling [86,87]. In the *in vitro* test, an effective decrease in ROS concentration was observed following treatment with low concentrations of HLJG0701-β, whereas CAT remained inactivated at low concentrations. However, activation of CAT may have occurred following treatment with 300 µg/mL HLJG0701-β, which was the highest concentration used in the test. Though CAT activation was not observed at lower concentrations, CAT tended to be activated as the concentration of the test substance increased, which suggests the emergence of an antioxidative effect at higher concentrations. Taken together, these results suggest that the neuroprotective effects of HLJG0701-β against H_2_O_2_-induced oxidative damage may involve the inhibition of ROS production.

The accumulation of Aβ_25-35_ plays an important role in the development of AD, by causing neuronal cell death [56,88]. In the present study, HLJG0701-β treatment significantly reduced Aβ_25-35_-induced neuronal damage in a concentration-dependent manner. Moreover, HLJG0701-β treatment inhibited H_2_O_2_-induced oxidative damage of the treated neuronal cells in a concentration-dependent manner. It is possible that HLJG0701-β treatment may reverse H_2_O_2_-induced oxidative damage by scavenging ROS, which is supported by the finding of increased ROS inhibition. Moreover, ginsenosides such as Rk1, a smaller molecular compound, exhibited higher antioxidant effects [42]. Therefore, it is likely that Rk1-enriched HLJG0701-β contributed to the higher ROS inhibition. In a previous study, the molecular mechanisms of the neuroprotective effects of ginsenosides were shown to include effects on both neuronal cells and neurotransmission. Ginsenosides such as Rg3 reduced the Aβ_25-35_-induced neuronal damage by up-regulating neprilysin (NEP) gene expression [41]. In another report, ginsenosides increased glutamate transporter-1 (GLT-1) expression and protein kinase B (PKB/Akt) levels in astrocytes [89]. Moreover, it has been argued that the total saponins present in ginseng extracts reduce H_2_O_2_-induced cell death in primary rat cortical astrocytes through the up-regulation of antioxidant systems, including glutathione S-transferase (GST) and heme oxygenase-1 (HO-1) [90].

Scopolamine, a non-selective muscarinic ACh receptor antagonist, is known to interfere with learning and short-term memory [91]. Animal models of scopolamine-induced memory impairment are quite useful to determine the effects of drugs on memory [92]. The animal behavior test conducted in this study is known to be the most effective test for evaluating short-term memory and reference memory, as it assesses the ability to learn about new spaces [92,93,94]. The Morris water maze test was used to show the effect of a substance in terms of delayed escape, time to reach the platform, and swimming time in the quadrant. The Y-maze test was used to determine whether a substance could increase the rate of spontaneous behavioral changes. C57BL/6 mice with scopolamine-induced short-term memory loss were divided into test groups to measure hippocampal-dependent spatial perception and learning ability in an underwater maze test, and instantaneous spatial cognition, an aspect of short-term memory, in a Y-maze test. HLJG0701-β administration in positive control-treated mice produced a significant increase in spontaneous alternation behaviors in a dose-dependent manner and a decrease in the total number of arm entries, thus indicating that HLJG0701-β may improve short-term and working memory. A similar result was also observed in the OVX + d-galactose-treated female mice. In the Morris water maze test, the mice treated with donepezil showed a rapid reduction in daily escape latency. HLJG0701-β administration at 250 and 500 mg/kg (treatment groups) and donepezil administration (positive control group) produced significant reductions in escape latency on day 5 compared to negative control treatment (*p <* 0.01). A similar result was observed in the OVX + d-galactose-treated female mice. Furthermore, HLJG0701-β treatment dramatically improved the swimming speed and time taken to cover the target quadrant compared to the negative control in both male and female mice. The transformation of ginsenosides in the cultured wild ginseng roots by the fermentation process may be a reason, as HLJG0701-β contains a higher concentration of small molecular compounds such as Rg3, Rk1 and Rg5. According to Kim et al. [61], ginsenosides such as Rg5 and Rh3 are capable of shortening escape latencies in the scopolamine-induced mice. Moreover, they found that ginsenosides including Rg5 and Rh3 significantly reversed the hippocampal brain-derived factor expression reduced by scopolamine. The results suggest that the HLJG0701-β extracts ameliorated the long-term memory impairments induced by scopolamine treatment in mice.

## 4. Materials and Methods

### 4.1. Fermentation Process

#### 4.1.1. Plant and Microbial Materials

Plant cell tissues were obtained from wild ginseng collected from Yanggu-gun, Gangwon-do, Korea. A plant tissue method was used to culture wild ginseng roots [95]. Prior to carrying out the present study, the different parameters of adventitious root regeneration, including types of culture media, concentration of basal salts, temperature, pH, inoculum density, elicitors, and carbon sources were optimized [52].

Briefly, adventitious roots (0.5–2.0 cm slices) were inoculated into a plant culture medium (67.1 g of Schenk and Hildebrandt (SH) medium mixed with 450 g sucrose and 15 L of purified water, with pH adjusted to 5.75 ± 0.10). The medium was placed into a bioreactor and sterilized at 121.0 °C for 60 min. The adventitious roots were inoculated in the medium under aseptic conditions and cultured for 8 weeks. The cultures were maintained at 15 °C and 30 °C during the day and night, respectively, with a 16:8 (L:D) photoperiod. Records of root growth began 5 weeks after initiation. The roots were treated with methyl jasmonate (250 μM) during the 3 week of cultivation. The roots were then harvested and used as materials for fermentation.

*Pediococcuspentosaceus* HLJG0702 (*P. pentosaceus*, KACC 81017BP) was used as the microorganism in this study. It was obtained from kimchi after separating, identifying, and sub culturing lactic acid bacteria [96]. A batch culture method was used to cultivate *P. pentosaceus* in microbial culture medium (55.25 g of de Man, Rogosa, and Sharpe, MRS) medium, mixed with 1 L of purified water with pH adjusted to 6.50 ± 0.05). The medium was placed into a fermenter and sterilized at 121.0 °C for 30 min. After cooling to room temperature, *P. pentosaceus* HLJG0702 was inoculated into the medium under aseptic conditions and cultured (30°C, 120–150 rpm) for more than 48 h. Thereafter, bacterial cells were recovered by centrifugation (2236R, Labogene, Seoul, Korea). The cells were then mixed with an anti-freezing agent (95% glycerol) and stored at −80 °C before use as the microbial material for fermentation.

#### 4.1.2. Fermentation of Cultured Wild Ginseng Roots

HLJG0701-β was prepared by mixing cultured wild ginseng root extract with *P. pentosaceus,* followed by fermentation with some modification [96]. To prepare the cultured wild ginseng root extract, the roots were extracted with 70% alcohol, filtered, and concentrated (60 Brix or more). The concentrate was then mixed with purified water at a ratio of 0.83% to 99.17% to prepare cultured wild ginseng root extract. The microbial material and MRS culture medium were mixed at a ratio of 0.02% to 99.98% and used for seed culture at 30 °C for 48 h or more. The seed culture solution and MRS culture medium were mixed at a ratio of 10% to 90% and used for the main culture at 30 °C for 48 h or more. *P. pentosaceus* was prepared by mixing cells harvested from centrifugation and purified water at a ratio of 0.31% to 99.69%. Then, the above-prepared cultured wild ginseng root extract and *P. pentosaceus* were mixed at a ratio of 80% to 20%, followed by fermentation at 120 ± 5 °C for 210 ± 10 min, filtration, and concentration (60 Brix or more) to prepare the final raw material, HLJG0701-β.

### 4.2. Analysis of Ginsenosides

#### 4.2.1. Reference Standard, Chemicals and Regents

Eight ginsenoside reference standards, Rb1, Rb2, Rb3, Rc, Rd, Rg3, Rg5, and Rk1, were purchased from Sigma Aldrich, Inc. (St. Louis, MO, USA). The purities of all reference standards were above 98%. High-performance liquid chromatography (HPLC)-grade acetonitrile (AcN), water, and methanol (MeOH) were purchased from Thermo Fisher Scientific (Waltham, MA, USA). All other reagents used in this study were of analytical grade. Ginsenosides were extracted three times from ginseng samples using a sonicator from Daihan Scientific Co., Ltd. (WUC-N47H, Wonju, Kangwon, Korea) and analyzed using a Thermo Fisher Scientific Ultimate 3000 Series (Waltham, MA, USA) liquid chromatography coupled with an ultraviolet detector, according to previous methods [50,97]. The ginsenoside standards Rb1 (1 mg/mL), Rb2 (1 mg/mL), Rb3 (1 mg/mL), Rc (1 mg/mL), Rd (1 mg/mL), Rk3 (1 mg/mL), Rg5 (1 mg/mL), and Rk1 (1 mg/mL) were dissolved in 70% MeOH and then diluted with 70% MeOH to obtain a series of mixture reference standard solutions of different concentrations. The solutions were filtered through a 0.2 µm syringe filter (Minisart SRP25, Sartorius Stedim Biotech, Göttingen, Germany) before HPLC analysis. Table 2 shows the function of the ginsenoside calibration curve and the correlation coefficient R.

#### 4.2.2. Sample Preparation and HPLC Analysis

Each test sample (2 g) was precisely weighed, added to a 50 mL flask, and completely dissolved after filling to 50 mL with 100% MeOH. The solution was filtered through a 0.2 μm syringe filter and used after diluting. The separation was based on a Capcell Pak C18 (250 × 4.6 mm, 5 μm) column (Osaka Soda Co., Ltd., Nishi, Osaka, Japan) at a temperature of 30 °C, analyzed with an injection volume of 10 μL at a flow rate of 1.0 mL/min, and detected at a wavelength of 203 nm. The binary gradient elution solvent consisted of water (A) and acetonitrile (B). The gradient elution program was as follows: 0–5 min, 80% A; 5–20 min, 80%–77% A; 20–25 min, 77%–70% A; 25–30 min, 70%–60%; 30–35 min, 60%–50% A; 35–65 min, 50%–15% A; 65–75 min, 15%–80% A. The peak areas corresponding to ginsenosides from the samples, with the same retention time as authentic ginsenosides (Rb1, Rc, Rb2, Rb3, Rd, Rg3, Rk1, Rg5), were integrated by comparison with an external standard calibration curve.

### 4.3. In Vitro Experiment

#### 4.3.1. Neuroprotective Effect

##### AChE Activity

The AChE activity of HLJG0701-β obtained from cultured wild ginseng roots was assessed using a colorimetric method [98]. Initially, the reaction mixture and 0.2 U/mL of AChE (ab138871, Abcam, Cambridge, UK) were added to each well of a microplate containing the test sample, followed by exposure to blocking light for 30 min at room temperature. Absorbance was scanned at 410 nm using a microplate reader (SpectramaxPLUS384, Molecular Device, San Jose, CA, USA). The measured values were corrected based on the absorbance (Abs) of the reaction mixture and test samples only, and the percentage AChE inhibition rate was calculated according to the following equation:

AChE inhibition rate (%) = (1 − OD_A_/OD_B_) × 100 (1)

where OD_A_: enzyme + substrate + absorbance after reaction of sample—control group absorbance; OD_B_: enzyme + absorbance after reaction of substrate—control group absorbance.

##### Cytotoxicity Assay

SH-SY5Y human neuroblastoma cells were used in this study to determine the effects of the test samples on cell viability. The cells were cultured with Dulbecco’s Modified Eagle’s Medium (DMEM) supplemented with 10% heat-inactivated fetal bovine serum (FBS), 2 mM l-glutamine, 100 U/mL penicillin, and 100 μg/mL streptomycin and incubated at 37 °C with 5% CO_2_. Cell Counting Kit-8 (CCK-8, Dojindo Molecular Technologies, Inc., Rockville, MD, USA) reagent was added to each well of a 96-well plate. After reacting for 2 h at room temperature, the absorbance was measured at 450 nm using a microplate reader. All samples were checked three times. The concentration of each material was set based on the concentration that did not affect cell survival. The methods were performed as previously described [99].

##### Nerve Cell Damage Assay

Aβ_25-35_ (Sigma Aldrich Inc., St. Louis, MO, USA) and H_2_O_2_ (Samchun Chemicals, Seoul, Korea) were used to induce nerve cell damage [100,101]. The neuroprotective effects of HLJG0701-β were then determined. Briefly, cells were seeded into a 96-well plate at a density of 5 × 10^4^ cells/well. After incubation at 37 °C in a CO_2_ incubator for 24 h, 5 µM Aβ_25-35_ or 100 µM H_2_O_2_ was applied to the cells along with HLJG0701-β for 24 h. The CCK-8 assay was then performed as described above. Cell survival (as assessed by the OD value) was taken to reflect the cell-protective effects of the test materials against nerve cell damage.

#### 4.3.2. Antioxidant Effect

##### ROS and CAT Activity

After plating 2 × 10^6^ cells into each well of a 6-well plate and incubating for 24 h, then treated with HLJG0701-β diluted in serum-free media at different concentrations for 24 h. As a positive control, 50 µM ascorbic acid (Vitamin C) was used. Oxidative stress was induced by adding 300 µM H_2_O_2_ and then incubating at 37 °C in a CO2 incubator for 4 h. The activity of ROS and CAT were measured according to the manufacturer’s instructions using a ROS detection assay kit (ab113851, Abcam, Cambridge, UK) and CAT assay kit (707002, Cayman Chemical, Ann Arbor, MI, USA).

### 4.4. In Vivo Experiment

#### 4.4.1. Experimental Animals

Male mice were used for the scopolamine-induced animal model and female mice were used for the d-galactose-induced aging animal model [70,102]. C57BL mice were purchased from Orient Bio (Seongnam, Gyeonggi, Korea). All animals were reared under controlled conditions (temperature of 22.1 ± 0.6 °C, relative humidity of 49.6 ± 4.6%, light intensity of 265 Lux, noise of 50.9 dB, and 12/12 h of light/dark cycle). Two animals were housed per polycarbonate cage. The animals had free access to experimental rodent feed (Envigo, Indianapolis, IN, USA) and reverse osmosis water. The test animals were acclimatized for 6 days before administration of the test substance. Only animals that were healthy during the acclimatization period were used for the test. To prepare the scopolamine-induced animal model, the test substance and positive control substance were administered once daily, 7 days/week for 7 weeks. During the period of the behavioral experiment, following 7 weeks of administration, the test substance or positive control was administered about 1 h before the start of the experiment. During the behavioral experiment, 1 mg/kg of scopolamine was injected intraperitoneally 30 min after administration of the test substance in all groups except the control group to induce memory loss.

To prepare the animal model of d-galactose-induced aging, the skin around the lumbar spine was disinfected following inhalation anesthesia with isoflurane, and a 2 cm cut was made around the lumbar spine. Both ovaries were removed. After extraction, the skin was sutured and disinfected. In the normal control group, all procedures except for ovarian extraction were performed in the same manner. To induce aging, d-galactose was administered subcutaneously at a dose of 100 mg/kg to animals whose ovaries were removed. The test substance and positive control substance were administered once daily, 7 days/week for 8 weeks. During the period of the behavioral experiment, after 8 weeks of administration, the test substance or positive control was administered about 1 h before the start of the experiment. The aging-inducing substance, test substance or positive control substance was administered once daily, 3 days/week for 6 weeks followed by 7 days/week for 2 weeks, for a total of 8 weeks. Scopolamine hydrobromide and d-galactose were purchased from Sigma Aldrich Inc. (St. Louis, MO, USA). Donepezil was obtained from Aurobindo Pharma Ltd. (Telangana, Karnataka, India).

#### 4.4.2. Treatments

A total of 60 animals were divided into six groups (10 animals per group): a control group, a scopolamine- or OVX + d-galactose-induced control group, a positive control (donepezil) group, and three HLJG0701-β groups. The control and scopolamine or OVX + d-galactose groups were administered sterile distilled water. The test groups were administered the test substances orally by gastric gavage. The test substance was HLJG0701-β. It was administered at doses of 125, 250, and 500 mg/kg. Donepezil, the positive control, was injected intraperitoneally at a dose of 5 mg/kg (Table 3).

#### 4.4.3. Morris Water Maze Task

The maze consisted of a stainless-steel circular pool with a diameter of 120 cm and a height of 50 cm. The pool was equally divided into four imaginary quadrants (NW, NE, SE, and SW); a visual cue of four unique shapes (square, triangle, circle, and star) was attached to the wall in each of the four quadrants. A transparent escape platform (6 cm in diameter and 29 cm in height) was located in the center of one quadrant (NE) and the pool was filled with water until the platform was submerged 1 cm below the surface. The water was made opaque with titanium oxide and maintained at a temperature of 22 ± 2 °C. All experimental conditions were maintained for the duration of the experimental period. During the two days before trial initiation, all animals were allowed to swim in the pool without a platform daily for 60 s in order to evaluate their locomotor activity and acclimation to the surrounding environmental conditions. Training sessions were conducted with two trials per day at an interval of 120 s for five consecutive days. Mice were semi-randomly released in one quadrant (without the platform) and the escape latency, the time it took the mouse to reach the hidden platform, was measured. The mice were allowed 60 s for exploration of the platform. It was acceptable for them to stay on the platform for 15 s once they found it. If the mice failed to locate the platform on their own, they were gently guided to it and allowed to remain on it for 15 s [92].

#### 4.4.4. Y-Maze Task

The Y-maze consisted of three white arms connected at 120° angles (A, B, and C arms; 35 cm long × 6 cm wide × 13 cm high). After placing a mouse on one arm and allowing it to move freely for 8 min, the order in which the mouse entered and exited each arm was recorded. If the tail of the animal fully entered an arm, it was considered to have entered that arm. The total number of times that a mouse accessed each arm was measured. The number of cases of successive entries to each arm (actual alternation, ABC, BCA, CAB, etc.) was measured and 1 point was given. Using this result, the alternation behavior was calculated as follows:

Alternation (%) = actual alternation/(total arm entries − 2) × 100(2)

The total number of arm entries was an indicator of the locomotor activity of the animals.

#### 4.4.5. AChE and ACh Contents in Brain Tissue

Immediately after the last behavioral experiment, the mice were euthanized and their brains, excluding the cerebellum, were extracted. These tissues were homogenized in 1 mL of phosphate-buffered saline (pH 7.4, Gibco) and centrifuged at 5000 rpm for 10 min at 4 °C. The supernatant was used to determine AChE and ACh levels. Protein concentration was analyzed using a Pierce™ Rapid Gold BCA Protein assay kit (Thermo Fisher Scientific, Waltham, MA, USA). The same amount of protein for all samples was then used to measure AChE and ACh content using commercial kits (Abcam, Cambridge, UK) according to the manufacturer’s instructions [103].

#### 4.4.6. MDA and CAT Contents in Blood

Blood samples collected from the abdominal aorta were incubated for 30 min at room temperature in a serum separating tube (BD Vacutainer, Plymouth, UK). Serum was obtained by centrifugation at 1500 rpm for 10 min [104].

MDA has frequently been used to measure lipid peroxidation. MDA assay was performed by determining the reaction of malonaldehyde with two molecules of 1-methyl-2-phenylindole at 45 °C. The reaction mixture consisted of 0.64 mL of 10.3 mmol/L 1-methyl-2-phenylindole, 0.2 mL of sample and 10 μL of 2 μg/mL butylated hydroxyltoluene. After mixing by vortex, 0.15 mL of 37% HCl was added. The mixture was incubated at 45 °C for 60 min and centrifuged at 15,000 rpm/min for 20 min. The cleared supernatant absorbance was determined at 586 nm. An MDA assay kit (ab118970, Cayman Chemical, Ann Arbor, MI, USA) was used for the analysis. For CAT activity, a CAT assay kit (707002, Cayman Chemical, Ann Arbor, MI, USA) was used for the analysis.

### 4.5. Statistics

In the *in vitro* tests used to measure the degree of AChE inhibition by the test substance, a test case in which the added substrate did not react with AChE was set as having an inhibition rate of 100%. The absorbance value of the group treated with sterile distilled water was taken to represent 0% AChE inhibition, assuming that both the enzyme and the substrate reacted. If the value was negative, it was set at 0. To measure the neuroprotective effect of the treatment based on ROS level and CAT activity, the results of the control, Aβ_25-35_, and H_2_O_2_ control groups were subjected to Student’s *t*-test. Differences among the Aβ_25-35_, H_2_O_2_ control, and test substance-administered groups were analyzed by one-way analysis of variance (ANOVA) to determine the significance of the findings and test for equal variance. If the significance and equal variance were recognized, Duncan’s test was performed. If the equal variance was not recognized, Dunnett’s T3-test was used. *p*-values of *<*0.05 indicated statistical significance. All statistical analyses were performed in accordance with the standard operating guidelines for statistical processing of this laboratory. All analyses were performed using SPSS 12.0 K (SPSS, Chicago, IL, USA), a widely used commercial statistical package. All *in vivo* test data are presented as the average ± SEM. Differences between the control and negative control groups were compared with an independent samples *t*-test. Differences between the negative control group and the HLJG0701-β administered group were analyzed for significance through a one-way ANOVA and tested for equal variance. In the one-way ANOVA, if significance and equal variance were recognized, Duncan’s test was performed. However, if the equal variance was not recognized, Dunnett’s T3-test was used. All statistical analyses were performed using IBM SPSS software (Armonk, NY, USA), with a *p*-value of less than 0.05 indicating statistical significance.

## 5. Conclusions

We established a culture technology that could artificially produce wild ginseng root through *in vitro* culture. Small-molecule ginsenosides in the test substance, HLJG0701-β, were produced by bioconversion through steaming and fermentation. The total ginsenoside content of cultured wild ginseng root was increased after methyl jasmonate treatment (elicitor), and HLJG0701-β was developed by improving the manufacturing process. To verify the functionality of the new raw material, HLJG0701-β, we determined its AChE inhibitory effect and neuroprotective effects against Aβ_25-35_ and H_2_O_2_. Based on the results of *in vitro* and *in vivo* evaluations, HLJG0701-β may be considered as a potent candidate for the inhibition of AChE activity and oxidative stress, and thus may be involved in improving memory deficit in mice. It also improved memory by increasing the activity of cholinergic nerves. The efficacious and safe dose of HLJG0701-β needs to be determined through future clinical trials. The results of this study suggest that HLJG0701-β can be developed as a raw material for healthy functional foods.

## Figures and Tables

**Table 1:** Ginsenoside contents in cultured wild ginseng root and HLJG0701-β (mean ± SD).

Treatment	Ginsenoside Contents (mg/g)								
	High Molecular Compounds					Small Molecular Compounds			Total
	Rb1	Rc	Rb2	Rb3	Rd	Rg3	Rk1	Rg5	
Cultured wild ginseng root	51.53 ± 1.34	38.16 ± 1.10	34.36 ± 1.26	8.10 ± 0.52	55.90 ± 0.85	N.D.^1^	N.D.	N.D.	188.06 ± 4.98
HLJG0701-β	9.26 ± 0.28	4.93 ± 0.41	6.36 ± 0.40	2.83 ± 0.35	11.26 ± 0.56	44.26 ± 1.02	15.93 ± 0.32	23.10 ± 0.59	117.96 ± 3.38

^1^ N.D.: not detected.

**Table 2:** Chemical structures and molecular of reference ginsenoside standards in this study. M.W., molecular weight.; glc,β-d-glucopyranosyl; arap,α-l-arabinopyranosyl; xyl, β-d-xylopyranosyl; araf, α-l-arabinofuranosyl.

Analyte	Formula	Side Chain	R_1_	R_2_	R_3_	R_4_	M.W.	Function	R-Squared
Rb1	C_54_H_92_O_23_	A1	glc(2-1)glc	H	Oglc(6-1)glc	CH_3_	1109.29	y = 0.0431x + 0.0123	R^2^ = 0.9995
Rc	C_53_H_90_O_22_	A1	glc(2-1)glc	H	Oglc(6-1)araf	CH_3_	1079.27	y = 0.0427x + 0.0568	R^2^ = 1.000
Rb2	C_53_H_90_O_22_	A1	glc(2-1)glc	H	Oglc(6-1)arap	CH_3_	1079.27	y = 0.0481x + 0.0197	R^2^ = 0.9988
Rb3	C_53_H_90_O_22_	A1	glc(2-1)glc	H	Oglc(6-1)xyl	CH_3_	1079.27	y = 0.0396x + 0.0875	R^2^ = 0.9999
Rd	C_48_H_82_O_18_	A1	glc(2-1)glc	H	Oglc(6-1)	CH_3_	947.15	y = 0.0503x + 0.0605	R^2^ = 1.000
Rg3	C_42_H_72_O_13_	A1	glc(2-1)glc	H	OH	CH_3_	785.01	y = 0.0626x + 0.0333	R^2^ = 1.000
Rk1	C_42_H_70_O_12_	A2	glc(2-1)glc	H	-	-	767.00	y = 0.1653x + 0.0546	R^2^ = 1.000
Rg5	C_42_H_70_O_12_	A3	glc(2-1)glc	H	-	-	767.00	y = 0.1911x + 0.1765	R^2^ = 0.999
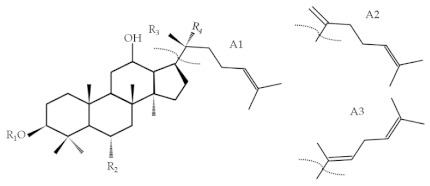									

**Table 3:** The scopolamine-, OVX+ d-galactose-induced mice model study of the test.

Model	Group	Number of Animals	Volume (mg/kg)	Dose (mg/kg)
Scopolamine Model (Male, 9 weeks old, 18.37–23.92 g)	Control	M1~10	5	0
	Scopolamine (1 mg/kg)	M11~20	5	0
	Scop + Donepezil (5 mg/kg)	M21~30	5	5
	Scop + HLJG0701-β	M31~40	5	125
		M41~50	5	250
		M51~60	5	500
Ovariectomized (OVX) +d-galactose Model (Female, 9 weeks old, 18.40–20.97 g)	Control	F1~10	5	0
	OVX + d-galactose (100 mg/kg)	F11~20	5	0
	OVX + d-galactose + Donepezil (5 mg/kg)	F21~30	5	5
	OVX + d-galactose + HLJG0701-β	F31~40	5	125
		F41~50	5	250
		F51~60	5	500

## Data Availability

The data presented in this study are available on request from the corresponding author.

## Sample Availability

Samples of the compounds are available from the authors.
